# The interaction of adverse childhood experiences and gender as risk factors for depression and anxiety disorders in US adults: a cross-sectional study

**DOI:** 10.1186/s12889-021-12058-z

**Published:** 2021-11-12

**Authors:** Robert C. Whitaker, Tracy Dearth-Wesley, Allison N. Herman, Amy E. Block, Mary Howard Holderness, Nicholas A. Waring, J. Michael Oakes

**Affiliations:** 1 Columbia-Bassett Program, Vagelos College of Physicians and Surgeons, Columbia University, New York, NY USA; 2 Columbia-Bassett Program, Bassett Medical Center, Cooperstown, NY USA; 3 Bassett Research Institute, Bassett Medical Center, Cooperstown, NY USA; 4 Department of Pediatrics, Vagelos College of Physicians and Surgeons, Columbia University, New York, NY USA; 5 Division of Epidemiology and Community Health, School of Public Health, University of Minnesota, Minneapolis, MN USA

**Keywords:** Depression, Anxiety, Adverse childhood experiences, Child abuse, Sex, Gender identity

## Abstract

**Background:**

Exposure to adverse childhood experiences (ACEs) and being female are distinct risk factors for having a major depressive episode (MDE) or an anxiety disorder (AD) in adulthood, but it is unclear whether these two risk factors are synergistic. The purpose of this study was to determine whether exposure to ACEs and being female are more than additive (synergistic) in their association with MDE and AD in US adults.

**Methods:**

We pooled cross-sectional survey data in the Midlife in the United States study from two nationally-representative cohorts of English-speaking US adults. Data from the first cohort were collected in 2004–2006 and from the second in 2011–2014. Data from both cohorts included the 12-month prevalence of MDE and AD (generalized anxiety disorder or panic disorder) assessed with the Composite International Diagnostic Interview Short Form, gender (here termed female and male), and the count of five categories of exposure to ACEs: physical, sexual, or emotional abuse; household alcohol or substance abuse; and parental separation or divorce.

**Results:**

Of the 5834 survey respondents, 4344 (74.5%) with complete data on ACEs were included in the analysis. Mean (SD) age was 54.1 (13.8) years and 53.9% were female. The prevalences of MDE, AD, and exposure to 3–5 categories of ACEs were 13.7, 10.0, and 12.5%, respectively. After adjusting for covariates (age, race, and current and childhood socioeconomic disadvantage), for those with both risk factors (female and 3–5 ACEs) the prevalence of MDE was 26.9%. This was 10.2% (95% CI: 1.8, 18.5%) higher than the expected prevalence based on the additive associations of the two risk factors. The adjusted prevalence of AD among females with 3–5 ACEs was 21.9%, which was 11.4% (95% CI: 4.0, 18.9%) higher than the expected prevalence.

**Conclusions:**

For both MDE and AD, there was synergy between the two risk factors of exposure to ACEs and being female. Identification and treatment of MDE and AD may benefit from understanding the mechanisms involved in the synergistic interaction of gender with ACEs.

**Supplementary Information:**

The online version contains supplementary material available at 10.1186/s12889-021-12058-z.

## Introduction

Adverse childhood experiences (ACEs), such as exposure to emotional, physical, or sexual abuse, are common [1] and associated with an increased risk of major depression and anxiety disorders in adulthood [2]. There are plausible socio-biological mechanisms to explain how the early life stress of ACEs contributes to the later risk of these disorders [3–6]. Separate from the risk factor of ACEs, females are at higher risk than males for these disorders [7–10]. Similar to ACEs, the mechanisms conferring risk for females appear to involve factors which are both social (gender) [11–14] and biological (sex) [15–17]. Gender and sex are distinct social and biological constructs, but they are inter-related, transcend binary designations, and act together in contributing to health outcomes [18]. For brevity, however, we henceforth only use the terms “gender” and “female/male.”

Although exposure to ACEs and being female are distinct causal risk factors for depression and anxiety, it is unclear whether these two factors are synergistic, or more than additive, in their associations. There are plausible socio-biological mechanisms of interaction between these risk factors. At different developmental stages, sex differences affect the brain in ways that can alter the stress response of the nervous, endocrine, and immune systems [19, 20]. For example, female sex hormones can enhance the neuro-immune response to ACEs, making females more susceptible to depression and anxiety [21–23]. The nervous, endocrine, and immune systems work together in response to physical and psychological threats, including violence, abandonment, and discrimination. It remains unclear whether any female/male differences in the bio-behavioral responses to such threats reflect evolutionary biology [24, 25] or gender socialization [26]. However, in sexist and patriarchal societies, many females experience chronic stress [27], and gender norms can lead to females holding distorted and negative perceptions and beliefs about their worth and functioning [28]. As in other preventable forms of discrimination, such as racism, chronic exposure to sexism, especially if experienced throughout development and in prior generations, can initiate neuro-endocrine-immune and behavioral processes that may directly cause anxiety and depression [29, 30]. Exposure to sexism, like racism, can also reduce the likelihood that the child will be able to buffer the effects of other adversities on their mental health. There is some evidence of synergy among ACEs on mental health disorders [31, 32], and sexism might potentially be viewed as an additional developmental trauma that acts synergistically with other ACEs.

Despite these plausible mechanisms of interaction between ACEs and gender, there is no clear epidemiologic evidence of synergy between these two risk factors in relation to the outcomes of major depression or anxiety disorders. Evaluating the presence of interaction as synergy requires a different approach to data analysis than testing for interaction as effect modification (or moderation) [33, 34]. Assessing synergy involves determining whether two putative causal risk factors when present together (being female and exposed to ACEs, in this instance) is associated with a greater observed risk of the outcome (major depression or anxiety disorders) than expected, where the expected risk is the sum of the two separate risks when each is present without the other. We identified seven studies [35–41] that examined the two risk factors using population samples, assessed major depression or anxiety disorders with measures based on the Diagnostic and Statistical Manual of Mental Disorders (DSM), and assessed exposure to at least three categories of ACEs. In five of these studies [36, 38–41] the authors performed a statistical test of interaction between ACEs and gender, but none found evidence of a significant interaction. Each study presented statistical tests of interaction on the multiplicative scale. Statistically significant and clinically meaningful interaction can be missed if one only tests for interaction on a multiplicative scale and not also on the additive scale, which tests for a significant departure from the additive associations of two risk factors [33]. In addition, none of these studies reported the prevalence of major depression or anxiety disorders for every risk strata defined by combinations of the two risk factors, as is recommended in evaluating interaction as synergy [42]. In two studies that did stratify by both risk factors [35, 37], the authors did not perform any statistical tests of interaction or estimate risk differences between strata. By employing methods that stratify the data by combinations of the two risk factors and identify significant departures from the additive associations [34], we can potentially identify synergy between ACEs and gender that may have been previously overlooked. This synergy has important implications for both the prevention and treatment of depression and anxiety disorders. Evidence of synergy would bring more attention to the possibility that sexism is a modifiable cause of these disorders that interacts with ACEs. Acting on that evidence in both prevention and treatment may help reduce the large burden of depression and anxiety among females that is attributable to ACEs [43].

Using data from a nationally-representative sample of US adults, we examined whether exposure to ACEs and being female are synergistic risk factors in their association with the 12-month prevalence of a major depressive episode (MDE) and an anxiety disorder (AD) (panic disorder and/or generalized anxiety disorder).

## Methods

### Study population and design

We used survey data from the Midlife in the United States (MIDUS) study [44], pooling data from two different MIDUS cohorts. Participants were recruited through random-digit-dialing, and the cohorts were designed to be representative of non-institutionalized, English-speaking adults living in the contiguous United States. We only included MIDUS participants who were recruited through random-digit-dialing sampling. Data were collected first by phone interview and then by mailed self-administered questionnaire (SAQ); the same survey items were used with both cohorts. The first cohort (*N* = 2257) was surveyed in 2004–2006 (MIDUS 2, M2) [45, 46], and the second cohort (*N* = 3577) in 2011–2014 (MIDUS Refresher, MR1) [47, 48]. For this cross-sectional analysis we combined data from both cohorts (*N* = 5834 [2257 + 3577]). Because the MIDUS data we used were de-identified and publicly available [49], our study did not require institutional review board approval.

### Measures

#### Depression and anxiety disorders

The 12-month prevalence of a major depressive episode (MDE), panic disorder (PD), and generalized anxiety disorder (GAD) were each assessed by phone interview using items from the Composite International Diagnostic Interview Short Form (CIDI-SF) [50–53]. We combined those with PD and/or GAD into one group called anxiety disorder (AD), and we assessed the 12-month prevalence of MDE and AD as our two primary outcomes.

#### Adverse childhood experiences

We assessed participants’ recalled exposure, before 18 years of age, to five categories of ACEs: emotional abuse, physical abuse, sexual abuse, household alcohol or substance abuse, and parental divorce or separation. We determined these exposures using MIDUS survey items that had wording similar to items used in the ACE module of the Behavioral Risk Factor Surveillance System (BRFSS) (**Table S1**) [54]. We did not assess the other three categories of ACEs in the BRFSS module (mental illness in the household, intimate partner violence, and incarcerated household member) because the MIDUS surveys did not contain similarly worded items. An ACE score was created by counting the number of categories of exposure (range 0–5). To facilitate clinical interpretation of our data and allow us to assess any non-linear relationship between the ACE score and MDE or AD, we analyzed the ACE score as a categorical variable with 4 levels: 0, 1, 2, and 3–5 categories of exposure to ACEs.

#### Gender

During the MIDUS recruitment phone screener, the available household member identified each of the other household members as either female or male, before a respondent was selected from each household to participate. The designation of female or male was confirmed with participants in subsequent surveys. However, sex assigned at birth *and* gender identity were not assessed separately [55, 56]. For brevity, we labeled this variable as “gender” rather than “gender-sex” and use the designations “female” and “male” that were the binary designations used with respondents during data collection. In selecting these terms, however, we mean to convey our understanding that the plausible causal mechanisms leading to MDE and AD, as discussed above, involve both gender and sex and cannot be easily separated in research on humans.

#### Covariates

We included four covariates in our analyses which were potential confounders: age, race (self-reported as White, Black, other), childhood socioeconomic disadvantage (SED), and current SED. We created the childhood SED score (range 0 to 6) [57–60] and a current SED score (range 0 to 8) [59], with higher scores reflecting greater SED (see **Supplementary Appendix** for a detailed description).

### Statistical analysis

Our analysis was restricted to the 4346 participants who returned the SAQ because it included the items needed to construct the ACE score. Two additional participants with missing items for the score were excluded, leaving 4344 (74.5%) for analysis. We applied the post-stratification weights developed by the MIDUS research team for participants who returned the SAQ. The weights aligned the distribution of the SAQ participants with the Current Population Survey of the US Census Bureau in terms of gender, race, age, education, and marital status. We used a significance threshold of *P* < .05 from 2-sided testing.

Logistic regression models were run separately for MDE and AD outcomes. Each model included variables for the ACE score (as 4 levels) and gender (male/female) along with the covariates. In models with all participants, we first estimated the independent associations of the ACE score and gender with each outcome. We then ran these regression models separately for males and females.

We evaluated additive interaction between the risk factors of ACEs and gender by following the recommendations outlined in the Strengthening the Reporting of Observational Studies in Epidemiology (STROBE) statement [42] and employing the method suggested by Knol and VanderWeele [34]. We first used regression-based margins, standardized to the distribution of covariates in the study population, to estimate covariate-adjusted (or standardized) prevalences (95% CI) of MDE and AD for each of the eight groups defined by gender and level of exposure to ACEs [61].

We then examined how the joint association of the two risk factors (assessed here as the adjusted prevalence of the mental health outcome associated with having both risk factors) differed from the sum of the separate associations of each risk factor in the absence of the other risk factor. We considered there to be evidence of socio-biologic synergy between ACEs and gender if there was a departure from the additive associations of the two risk factors [33]. This was determined by an interaction contrast value > 0 with a 95% confidence interval (CI) excluding 0. The interaction contrast was calculated as the difference between the adjusted prevalence (probability) of the outcome (MDE or AD) for those with both risk factors (i.e., females with a given level of ACEs [P_11_]) and the expected prevalence. The expected prevalence was calculated as the sum of the adjusted prevalences associated with each separate risk factor (P_10_ + P_01_) minus the adjusted prevalence associated with having neither risk factor (i.e., males with no ACEs [P_00_]). In secondary analyses, the same analytic approach was used to examine synergy between gender and the five specific ACEs rather than the ACE score. We also tested for departure from multiplicative associations using the Wald test to examine model fit after adding interaction terms to the logistic models.

## Results

Of the 4344 included in the analysis, 53.9% were female, 85.1% were White. At the time of data collection for each cohort, those included in the analysis ranged in age from 25 to 84 years, and their mean (SD) age was 54.1 (13.8) years (Table 1). Those excluded due to missing responses (*n* = 1490) tended to be younger, less educated, and more often male (**Table S2**). The 12-month prevalences of MDE and AD were 13.7 and 10.0%, respectively, in our analytic sample, which were similar to the prevalences among those not included in the analysis (**Table S2**).

**Table 1:** Participant Characteristics

	All (***N*** = 4344)		Males (***n*** = 2009)		Females (***n*** = 2335)		
Characteristic^**a**^	No.	% (95% CI)^**a**^	No.	% (95% CI)^**a**^	No.	% (95% CI)^**a**^	***P*** Value^**b**^
Age, years^c^							
< 30	130	4.5 (3.7, 5.5)	63	5.7 (4.3, 7.7)	67	3.4 (2.6, 4.5)	
30–39	633	17.1 (15.8, 18.6)	282	15.9 (14.0, 18.1)	351	18.2 (16.3, 20.2)	
40–49	882	23.9 (22.4, 25.4)	403	24.2 (21.9, 26.7)	479	23.6 (21.6, 25.6)	.054
50–59	1003	24.6 (23.1, 26.1)	452	24.4 (22.2, 26.7)	551	24.7 (22.9, 26.7)	
60–69	1013	18.3 (17.1, 19.5)	485	18.3 (16.6, 20.1)	528	18.3 (16.7, 19.9)	
≥ 70	683	11.7 (10.7, 12.7)	324	11.5 (10.2, 13.0)	359	11.8 (10.6, 13.2)	
Race							
White	3745	85.1 (83.7, 86.3)	1779	86.2 (84.0, 88.1)	1966	84.1 (82.4, 85.7)	.070
Black	250	6.9 (5.9, 8.0)	76	5.6 (4.2, 7.5)	174	8.0 (6.8, 9.3)	
Other	325	8.0 (7.2, 9.0)	146	8.2 (6.8, 9.7)	179	8.0 (6.8, 9.3)	
Current SED score^d^							
0–1	809	13.5 (12.6, 14.5)	449	15.1 (13.7, 16.7)	360	12.1 (10.9, 13.5)	
2–3	1257	25.5 (24.1, 26.9)	611	25.6 (23.6, 27.8)	646	25.4 (23.6, 27.3)	.065
4–5	1298	31.5 (29.9, 33.2)	573	31.1 (28.7, 33.6)	725	31.9 (29.8, 34.1)	
6–8	940	29.5 (27.7, 31.3)	356	28.2 (25.4, 31.0)	584	30.6 (28.4, 32.9)	
Childhood SED score^e^							
0	853	17.9 (16.7, 19.2)	421	18.8 (17.0, 20.9)	432	17.2 (15.6, 18.9)	
1	999	21.8 (20.4, 23.2)	467	21.8 (19.7, 24.0)	532	21.7 (19.9, 23.6)	.729
2	1165	28.0 (26.4, 29.6)	525	27.5 (25.2, 30.0)	640	28.3 (26.3, 30.4)	
3	831	20.2 (18.8, 21.7)	379	20.3 (18.2, 22.6)	452	20.2 (18.4, 22.1)	
4–6	489	12.1 (11.0, 13.3)	213	11.6 (9.9, 13.4)	276	12.6 (11.1, 14.2)	
ACE score^f^							
0	1955	42.7 (41.0, 44.4)	946	43.3 (40.7, 45.9)	1009	42.2 (40.0, 44.5)	
1	1141	26.6 (25.0, 28.2)	515	26.6 (24.3, 29.1)	626	26.5 (24.6, 28.6)	.542
2	768	18.2 (16.9, 19.6)	351	18.5 (16.5, 20.7)	417	17.9 (16.2, 19.7)	
3–5	480	12.5 (11.4, 13.8)	197	11.6 (9.9, 13.5)	283	13.3 (11.8, 15.1)	
Major depressive episode							
Yes	521	13.7 (12.5, 15.0)	156	9.0 (7.5, 10.8)	365	17.8 (16.0, 19.7)	
No	3823	86.3 (85.0, 87.5)	1853	91.0 (89.2, 92.5)	1970	82.2 (80.3, 84.0)	<.001
Anxiety disorder							
Yes	372	10.0 (8.9, 11.1)	97	6.1 (4.8, 7.7)	275	13.2 (11.7, 14.9)	
No	3972	90.0 (88.9, 91.1)	1912	93.9 (92.3, 95.2)	2060	86.8 (85.1, 88.3)	<.001

Note: SED = socioeconomic disadvantage, ACE = adverse childhood experience; MIDUS = Midlife in the United States study

^a^No. and % (95% CI) = unweighted n and weighted percentages (95% CI) of sample. Percentages may not add to 100 due to rounding. Participants were missing data on covariates as follows: race (24 cases, 8 males and 16 females), childhood socioeconomic disadvantage score (7 cases, 4 males and 3 females), and current socioeconomic disadvantage score (40 cases, 20 males and 20 females)

^b^*P* value is for chi-square test assessing differences between males and females in the weighted proportion of participants at each level of a participant characteristic

^c^The combined sample mean (SD) = 54.1 (13.8) years

^d^Score based on 4 variables (highest level of education, perceived financial situation, enough money to meet needs, and difficulty paying monthly bills). Higher score (possible range 0–8) is more disadvantage

^e^Score based on 3 variables (welfare receipt and duration, financial status relative to others, and parental education). Higher score (possible range 0–6) is more disadvantage

^f^Score based on exposure to 5 categories of adverse childhood experiences (emotional abuse, physical abuse, sexual abuse, parental separation or divorce, and household alcohol or substance abuse)

In our analytic sample, MDE and AD were more common in females. Among those with AD, 54.2% reported MDE; among those with MDE, 39.2% reported AD (**Table S3**). The prevalences of exposure to 0, 1, 2, 3, 4, and 5 categories of ACEs were 42.7, 26.6, 18.2, 8.9, 3.3, and 0.3%, respectively. The distribution of ACE scores was not significantly different between males and females, with 56.7% of males and 57.8% of females reporting exposure to one or more categories of ACEs. However, childhood sexual abuse was reported more often by females (9.6% vs. 2.3%) and physical abuse more often by males (23.6% vs 18.5%) (**Table S4**). Among those reporting exposure to a given ACE, the majority reported experiencing another category of ACE. For example, another category of exposure to ACEs was reported by 80.9% of those reporting sexual abuse and 85.4% of those reporting physical abuse.

### Association of ACEs and gender with depression and anxiety disorders

In covariate-adjusted logistic regression models containing gender and ACE score, both risk factors were significantly associated with MDE and AD (Table 2). Compared to males, the adjusted odds of females experiencing MDE and AD in the prior 12 months were 2.24 (95% CI: 1.74, 2.87) and 2.40 (95% CI: 1.79, 3.22), respectively. There was also a graded association between the ACE score and adjusted odds of MDE and AD. These graded associations between the ACE score and both MDE and AD were stronger for females than males (Table 3). For example, the adjusted odds of MDE associated with reporting 3–5 categories of ACEs (compared to none) were 2.71 (95% CI: 1.80, 4.08) for females and 1.76 (95% CI: 0.96, 3.23) for males. Similarly, the adjusted odds of AD associated with reporting 3–5 categories of ACEs were 3.92 (95% CI: 2.48, 6.20) for females and 1.73 (95% CI: 0.78, 3.83) for males.

**Table 2:** Odds of Major Depressive Episode and Anxiety Disorder Associated with Two Risk Factors: Adverse Childhood Experience Score and Gender

	Prevalence of Disorder, Proportion (%)^**a**^	Model 1^**b**^	Model 2^**c**^	
Risk Factor		Odds Ratio (95% CI)	Odds Ratio (95% CI)	***P*** Value^**d**^
**Major Depressive Episode**^**e**^				
ACE score				
0	158/1955 (9.2)	Reference	Reference	
1	120/1141 (11.7)	1.30 (0.97, 1.74)	1.16 (0.86, 1.57)	
2	129/768 (19.2)	2.38 (1.79, 3.18)	2.01 (1.49, 2.72)	<.001
3–5	114/480 (25.5)	3.34 (2.45, 4.56)	2.34 (1.67, 3.28)	
Gender				
Males	156/2009 (9.0)	Reference	Reference	
Females	365/2335 (17.8)	2.18 (1.72, 2.78)	2.24 (1.74, 2.87)	<.001
**Anxiety Disorder**^**f**^				
ACE score				
0	95/1955 (5.5)	Reference	Reference	
1	89/1141 (9.6)	1.84 (1.30, 2.61)	1.70 (1.19, 2.44)	
2	101/768 (13.8)	2.82 (2.01, 3.95)	2.45 (1.72, 3.49)	<.001
3–5	87/480 (20.3)	4.38 (3.05, 6.27)	3.05 (2.06, 4.51)	
Gender				
Males	97/2009 (6.1)	Reference	Reference	
Females	275/2335 (13.2)	2.33 (1.74, 3.11)	2.40 (1.79, 3.22)	<.001

^a^Prevalence is unadjusted. Proportion = number with the mental health disorder/ number in the group defined by adverse childhood experience (ACE) score (count of 5 categories of exposure to ACEs as 4 levels) or gender (male or female). All numbers unweighted. Percentage = weighted 12-month prevalence of major depressive episode or anxiety disorder

^b^Model 1 is logistic regression model with the mental health disorder (major depressive episode or anxiety disorder) as the dependent variable and ACE score (as 4 levels) and gender as independent variables (*N* = 4344)

^c^Model 2 is logistic regression model with the mental health disorder (major depressive episode or anxiety disorder) as the dependent variable and ACE score, gender, and 4 covariates (age, race, childhood socioeconomic disadvantage [SED] and current SED) as independent variables. *N* = 4275 after a listwise deletion of 69 participants (31 males and 38 females), who were missing data on race, childhood SED, or current SED

^d^*P* value for the Wald test, which was used to assess whether the addition of the ACE score or gender significantly improved the model fit over a model with 4 covariates and the other risk factor (ACE score or gender)

^e^Wald test was used to assess the addition of three gender x ACE interaction terms to Model 2: F(3, 4272) = 1.22; *P* = .302 (assessing interaction in a multiplicative model)

^f^Wald test was used to assess the addition of three gender x ACE interaction terms to Model 2: F(3, 4272) = 1.17; *P* = .318 (assessing interaction in a multiplicative model)

**Table 3:** Odds of Major Depressive Episode and Anxiety Disorder Associated with Adverse Childhood Experience Score, Stratified by Gender

	Prevalence of Disorder, Proportion (%)^**a**^	Model 1^**b**^	Model 2^**c**^	
Risk Factor		Odds Ratio (95% CI)	Odds Ratio (95% CI)	***P*** Value^**d**^
**MDE -- Males**				
ACE score^e^				
0	55/946 (6.9)	Reference	Reference	
1	38/515 (8.1)	1.20 (0.70, 2.04)	1.02 (0.59, 1.77)	
2	34/351 (11.4)	1.74 (1.02, 2.98)	1.38 (0.78, 2.44)	.254
3–5	29/197 (15.2)	2.42 (1.37, 4.28)	1.76 (0.96, 3.23)	
**MDE -- Females**				
ACE score				
0	103/1009 (11.3)	Reference	Reference	
1	82/626 (14.7)	1.36 (0.96, 1.91)	1.24 (0.86, 1.79)	
2	95/417 (26.2)	2.79 (1.98, 3.93)	2.44 (1.70, 3.49)	<.001
3–5	85/283 (33.2)	3.91 (2.69, 5.69)	2.71 (1.80, 4.08)	
**AD -- Males**				
ACE score				
0	32/946 (4.2)	Reference	Reference	
1	23/515 (5.9)	1.41 (0.72, 2.76)	1.25 (0.63, 2.47)	
2	26/351 (8.3)	2.04 (1.09, 3.84)	1.79 (0.94, 3.41)	.273
3–5	16/197 (10.1)	2.54 (1.20, 5.34)	1.73 (0.78, 3.83)	
**AD -- Females**				
ACE score				
0	63/1009 (6.5)	Reference	Reference	
1	66/626 (12.8)	2.10 (1.41, 3.14)	1.98 (1.31, 3.01)	
2	75/417 (18.7)	3.30 (2.22, 4.88)	2.89 (1.90, 4.39)	<.001
3–5	71/283 (27.9)	5.56 (3.67, 8.42)	3.92 (2.48, 6.20)	

^a^Prevalence is unadjusted. Proportion = number with the mental health disorder/ number in the group defined by adverse childhood experience (ACE) score (count of 5 categories of exposure to ACEs as 4 levels). All numbers unweighted. Percentage = weighted 12-month prevalence of major depressive episode or anxiety disorder

^b^Model 1 is logistic regression model with the mental health disorder (major depressive episode or anxiety disorder) as the dependent variable and ACE score (as 4 levels) as the independent variable. For males, *N* = 2009 and for females *N* = 2335

^c^Model 2 is logistic regression model with the mental health disorder (major depressive episode or anxiety disorder) as the dependent variable and ACE score and 4 covariates (age, race, childhood socioeconomic disadvantage [SED] and current SED) as independent variables. For males, *N* = 1978 after a listwise deletion of 31 participants who were missing data on race, childhood SED, or current SED. For females, *N* = 2297 after a listwise deletion of 38 participants who were missing data on race, childhood SED, or current SED

^d^*P* value for the Wald test, which was used to assess whether the addition of the ACE score significantly improved the model fit over a model with 4 covariates

### Interaction between gender and ACEs

**Fig. 1:**
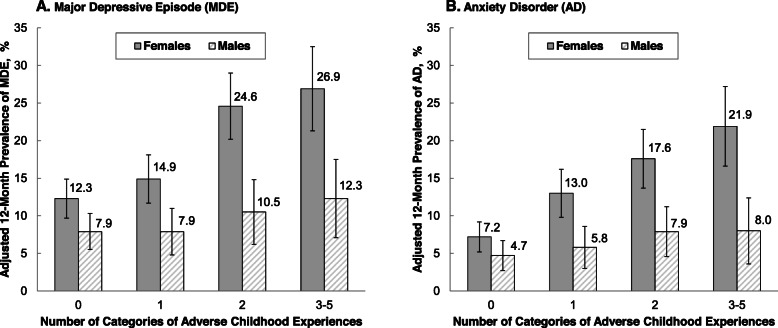
Adjusted Prevalence of Major Depressive Episode and Anxiety Disorder by Number of Categories of Adverse Childhood Experiences and Gender. Bars represent the adjusted 12-month prevalences of major depressive episode (MDE) and anxiety disorder (AD), and the uncertainty bars extend to the upper and lower bounds of the 95% confidence interval (CI). The adjusted prevalences (95% CI) were standardized to the distribution of covariates in the entire study population: age, race, childhood socioeconomic disadvantage, and current socioeconomic disadvantage

For both MDE and AD, there was evidence of significant additive interaction between the risk factors of gender (being female) and exposure to ACEs (Fig. 1 and Table 4). For example, the adjusted prevalence of MDE was 26.9% among females with 3–5 ACEs. This adjusted prevalence represents the joint association of the two risk factors (being female and 3–5 ACEs) on MDE. This was 10.2% (95% CI: 1.8, 18.5%) higher than the prevalence that would be expected based on the sum of the associations of the two risk factors considered separately—the interaction contrast (95% CI) shown in Table 4. The adjusted prevalence of MDE for females with 2 ACEs was 24.6%, which was 9.8% (95% CI: 2.8, 16.7%) higher than the sum of the separate associations of the risk factors. For the outcome of AD, the adjusted prevalences for females with 3–5 ACEs and 2 ACEs were 21.9 and 17.6%, respectively, which were 11.4% (95% CI: 4.0, 18.9%) and 7.2% (95% CI: 1.4, 12.9%) higher, respectively, than the sum of the separate associations. When the three interaction terms for gender (0 = M and 1 = F) by ACE score (categories of 0, 1, 2, 3–5) were added as a group to models with gender, ACE score, and the covariates, the addition of these interaction terms significantly improved the prediction of the outcomes of MDE and AD in the additive model (*P* = .011 and *P* = .004, respectively) but not in the multiplicative (logistic) model (*P* = .302 and *P* = .318, respectively). Evidence for synergy was also found when we re-examined the data three ways: unweighted, separately for those in each cohort, and without adjusting for current SED.

**Table 4:** Additive Interaction of Adverse Childhood Experiences with Gender on Major Depressive Episode and Anxiety Disorder

	Number of Categories of Adverse Childhood Experiences						
	0	1		2		3–5	
**Gender**	Adjusted Prevalence % (95% CI)^a^	Adjusted Prevalence % (95% CI)^a^	Interaction Contrast (95% CI)^b^ P_11_-P_10_- P_01_+ P_00_	Adjusted Prevalence % (95% CI)^a^	Interaction Contrast (95% CI)^b^ P_11_-P_10_- P_01_+ P_00_	Adjusted Prevalence % (95% CI)^a^	Interaction Contrast (95% CI)^b^ P_11_-P_10_- P_01_+ P_00_
**Major Depressive Episode**^c^							
Males	P_00_ = 7.9 (5.6, 10.3)	P_01_ = 7.9 (4.8, 10.9)	14.9–12.3–7.9 + 7.9 = 2.6 (−3.0, 8.1)	P_01_ = 10.5 (6.2, 14.7)	24.6–12.3–10.5 + 7.9 = 9.8 (2.8, 16.7)	P_01_ = 12.3 (7.1, 17.5)	26.9–12.3–12.3 + 7.9 = 10.2 (1.8, 18.5)
Females	P_10_ = 12.3 (9.8, 14.9)	P_11_ = 14.9 (11.7, 18.1)		P_11_ = 24.6 (20.3, 29.0)		P_11_ = 26.9 (21.3, 32.5)	
**Anxiety Disorder**^d^							
Males	P_00_ = 4.7 (2.8, 6.7)	P_01_ = 5.8 (3.0, 8.6)	13.0–7.2–5.8 + 4.7 = 4.7 (−0.3, 9.7)	P_01_ = 7.9 (4.6, 11.2)	17.6–7.2– 7.9 + 4.7 = 7.2 (1.4, 12.9)	P_01_ = 8.0 (3.6, 12.4)	21.9–7.2–8.0 + 4.7 = 11.4 (4.0, 18.9)
Females	P_10_ = 7.2 (5.3, 9.2)	P_11_ = 13.0 (9.8, 16.2)		P_11_ = 17.6 (13.7, 21.4)		(P_11_) = 21.9 (16.6, 27.2)	

^a^Weighted and adjusted 12-month prevalence of the outcome (major depressive episode or anxiety disorder) in the group defined by gender and number of categories adverse childhood experiences (ACEs). The adjusted prevalences (95% CI) were standardized to the distribution of covariates in the entire study population: age, race, childhood socioeconomic disadvantage (SED) and current SED

^b^The interaction contrast (95% CI) is calculated using the following formula: (P_11_-P_00_)-[(P_10_- P_00_) + (P_01_- P_00_)]. This formula can be simplified to: P_11_-P_10_- P_01_+ P_00._ In the formula, P represents the covariate-adjusted prevalence of the outcome and the subscripts 0 and 1 represent the groups defined by the presence (1) or absence (0) of one of the two risk factors. For example, P_00_ = male (0) with no ACEs (0) and P_11_ = female (1) with ACEs (1). Reported contrast values may vary from calculated values due to rounding. Contrast value > 0 (and 95% CI that excludes 0) is interpreted as more than additive interaction between the two risk factors

^c^Addition of all three interaction terms to the additive model: F(3, 4272) = 3.74; *P* = .011

^d^Addition of all three interaction terms to the additive model: F(3, 4272) = 4.47, *P* = .004

In secondary analyses, the associations of the five specific ACEs with MDE and AD (**Tables S5 and S6**) were similar to the analogous associations we found with the ACE score, with some exceptions. We also found significant additive interactions between gender and specific ACEs (**Figs. S1 and S2** and **Table S7**).

## Discussion

In a cross-sectional analysis of survey data from US adults, we found that exposure to ACEs and being female are synergistic risk factors for a current MDE or an AD. This means that the risk associated with the combination of these two factors is greater than the sum of the independent risks. For example, the prevalence of AD among females with 3–5 ACEs was more than twice as high as the expected prevalence (21.9% vs. 10.5%) based on the sum of the two separate risk factors. Synergy also means that exposure to ACEs poses a greater risk for depression and anxiety in females than in males. If over one-third of all cases of adult depression and anxiety disorders are due to ACEs [43], then this proportion is even greater for females.

### Research in context

Other population-based studies have not demonstrated a significant interaction between ACEs and gender as risk factors for adult depression and anxiety disorders [36, 38–41]. However, these studies may not have detected synergy between the two risk factors because the investigators tested for departure from multiplicative associations rather than from additive associations, as we did [33]. These studies did not document the separate associations of the two risk factors and their joint association using one reference category [42], so it is not possible to evaluate whether the data from these studies, like our own, showed evidence of significant departure from additive associations (synergy) without a significant departure from multiplicative associations. Chapman and colleagues, using data from 9460 members of the San Diego (CA) Kaiser Permanente health plan participating in the Adverse Childhood Experiences Study, reported the association between the ACE score (0–7) and current depression separately by gender [37]. The unadjusted prevalence data in that report suggest synergy between exposure to ACEs and being female, but interaction was not formally tested. Afifi and colleagues, using data from 5692 participants in the 2001–2003 US National Comorbidity Survey Replication, also stratified their analyses by gender but not in a manner that permitted evaluation of possible synergy [35].

### Limitations

From this single, cross-sectional study we cannot make causal inferences about the association between ACEs and mental health. However, the aggregate evidence from many studies of varying designs suggests that exposure to ACEs and being female are each distinct causal risk factors for adult depression or anxiety disorders. Recall bias, common-rater bias, and residual confounding are limitations of a cross-sectional design. Recall bias, in particular, is a well-studied methodological challenge when examining the association of ACEs and mental health in populations [62, 63]. This challenge arises, in part, from the inherent subjectivity of one’s experience of adverse events, particularly those that occur in childhood. However, we are not aware of evidence that recall bias affects males and females to a different degree [64] or in a manner that would alter our conclusions about synergy between exposure to ACEs and being female.

Apart from recall bias, misclassification bias could have resulted from assessing only five categories ACEs. There are known limitations of the most widely used measure of ACEs [65], which includes the 10 categories of exposure used in the Adverse Childhood Experiences Study [66] and later implemented in BRFSS [54]. For example, some have suggested expanding the list of ACEs to include measures of childhood exposure to socioeconomic deprivation and inequity [67], which we analyzed as a potential confounder. However, we are not aware of any evidence that other approaches to generating an ACEs score would have altered our findings about synergy between exposure to ACEs and being female. Using categories of ACEs that were part of the original ACE score increased the comparability of our findings to other studies that examined the interaction between ACEs and gender as risk factors for adult depression and anxiety disorders [36, 38–41]. In addition, by limiting the ACE score to the five categories of ACEs that were assessed in MIDUS with wording similar to BRFSS, we were able to show that the level of ACE exposure in the MIDUS sample was comparable to that in BRFSS (**Table S2**).

Misclassification bias may have also resulted in assessing the outcome, because we used the CIDI-SF, which is an abbreviated assessment based on DSM-IV diagnostic criteria [68]. Gender and sex were not measured as separate constructs, which may conflate putative causal mechanisms. However, this epidemiologic study was designed to evaluate the presence of synergy between exposure to ACEs and being female and not to determine the mechanisms. Finally, our use of sampling weights permit inference to the US population but use of the weights generally increases variance estimates and does not assure generalizability.

### Implications for research and practice

The scientific and social contexts that inform our understanding of ACEs [65, 67, 69], gender [70], and depression and anxiety [71, 72] are all changing rapidly, and these changes will continue to alter how these constructs are measured in research and practice. Researchers and clinicians should focus not only on how ACEs, even as traditionally assessed, interact with gender, but also how ACEs interact with other socially-determined constructs, such as race, which can cause trauma [29, 30, 73]. Additionally, future research should collect data on experiences of sexism to better understand the mechanisms linking gender, ACEs, and mental health.

The effectiveness of treating depression and anxiety disorders in girls and women might be improved by treatments that address the joint impacts of developmental trauma and sexism. Although our study did not include data on sexism, our findings on synergy are consistent with the possibility that the stresses of sexism can be reinforced, exacerbated, or amplified by other childhood traumas in a way that makes the risk of these traumas and being female more than additive. Therefore, treatments for depression and anxiety disorders in girls and women may be unsuccessful if they focus only or primarily on symptom management, with either medications or behavioral therapy. Broader training is needed in psychology, social work, and psychiatry on treatments that address the underlying and synergistic interaction between ACEs and gender, including sexism [74].

Addressing rigid gender norms and sexism may benefit the health of the entire population. For girls and women, reducing the harmful effects of sexism on psychophysiology might prevent the added exposure to ACEs from resulting in depression and anxiety disorders. Regardless of one’s gender identity, rigid gender norms can place harmful constraints on emotional expression, behavior, and social roles, which can negatively impact mental health [28]. Finally, the mental health consequences of potentially traumatic experiences can be worsened by gender stereotypes that determine and constrain the “acceptable” ways of managing those experiences [75].

In conclusion, this study, conducted using data collected in a nationally-representative sample of US adults, provides evidence that ACEs and being female are synergistic (more than additive) risk factors for depression and anxiety disorders. Beyond ongoing efforts to prevent ACEs, this study points to the potential to improve the prevention and treatment of these common mental health disorders by addressing sexism as a potentially modifiable traumatic experience. Preventing sexism will require a recognition that it can occur alongside other types of discrimination, such as racism, with which it interacts [76] and which are also transmitted socially across generations [77].

## Supplementary Information

**Additional file 1.** Supplementary Appendix.

## Data Availability

The data that support the findings of this study are openly available at the Inter-University Consortium for Political and Social Research: MIDUS I data at 10.3886/ICPSR02760.v18; MIDUS II at 10.3886/ICPSR04652.v7; and MIDUS Refresher at 10.3886/ICPSR36532.v3.
