# Supplementary material for: The interaction of adverse childhood experiences and gender as risk factors for depression and anxiety disorders in US adults: a cross-sectional study
Source: BMC Public Health. 2021 Nov 12;21:2078. doi: 10.1186/s12889-021-12058-z (PMC8590371; doi:10.1186/s12889-021-12058-z)
Supplement: Supplementary file 1 — Additional file 1. Supplementary Appendix. [file 12889_2021_12058_MOESM1_ESM.docx]

**Whitaker RC, Dearth-Wesley T, Herman AN, Block AE, Holderness MH, Waring NA, Oakes JM. The Interaction of Adverse Childhood Experiences and Gender as Risk Factors for Depression and Anxiety Disorders in US Adults: a Cross-Sectional Study**

Table of Contents for Online Supplement

| **Supplementary Appendix** | Description of Measures of Socioeconomic Disadvantage | p. 2 |
| --- | --- | --- |
| **Table S1** | Items Used to Create the Adverse Childhood Experience Score and Comparison Items Used in the Behavioral Risk Factor Surveillance System Adverse Childhood Experiences Module | p. 3-5 |
| **Table S2** | Comparison of Demographic Characteristics of MIDUS Participants Included and Not Included in the Analyses | p. 6 |
| **Table S3** | Relationships between Mental Health Conditions | p. 7 |
| **Table S4** | Relationships between Categories of Adverse Childhood Experiences | p. 8 |
| **Table S5** | Odds of Major Depressive Episode and Anxiety Disorder Associated with Two Risk Factors: Specific Adverse Childhood Experiences and Gender | p. 9-10 |
| **Table S6** | Odds of Major Depressive Episode and Anxiety Disorder Associated with Specific Adverse Childhood Experiences, Stratified by Gender | p. 11-12 |
| **Table S7** | Additive Interaction of Specific Adverse Childhood Experiences with Gender on Major Depressive Episode and Anxiety Disorder | p. 13 |
| **Figure S1** | Adjusted Prevalence of Major Depressive Episode by Specific Adverse Childhood Experiences and Gender | p. 14 |
| **Figure S2** | Adjusted Prevalence of Anxiety Disorder by Specific Adverse Childhood Experiences and Gender | p. 15 |
| **Supplementary References** | Online-Only Supplemental Material References | p. 16 |

This supplementary material has been provided by the authors to give readers additional information about their work.

**Supplementary Appendix. Description of Measures of Socioeconomic Disadvantage (SED)**

**Childhood SED**

We created a childhood SED score using retrospective reports in three areas: welfare receipt and duration, financial status relative to others, and parental education.^1-4^ For the MIDUS 2 cohort, we used responses collected during MIDUS 1 (1995-1996) because the items used to create the score were not asked at MIDUS 2. Respondents were asked whether their family was ever on welfare (excluding health insurance or unemployment benefits) for a period of six months or longer during their childhood or adolescence (Yes/No) and, if yes, whether that assistance was during “all, most, some, or only a little” of their childhood and adolescence. Reponses were combined and coded as: no (0); yes, some or only a little (1); and yes, all or most (2). Respondents were asked on a scale whether their family was better or worse off financially than the average family at that time. Responses were re-coded as: a lot better off, somewhat better off, or a little better off (0); same as the average family (1); and a little worse off, somewhat worse off, or a lot worse off (2). Separate questions were asked about the highest level of education for each parent, and responses were combined and coded as: some college or higher for at least one parent (0), high school graduate or GED for at least one parent (1), and less than high school for both parents (2). We summed values across the three derived variables to create the childhood SED score, with higher scores (range 0 to 6) reflecting greater childhood SED.

**Current SED**

We created a current SED score from four variables, one pertaining to the participant’s educational attainment and three to their current financial situation.^3^ Highest level of education was coded as: bachelor’s degree or higher (0); some college or an associate’s degree (1); and high school graduate, GED, or less (2). Participants ranked their perceived financial situation on a scale of 0 to 10 from “the worst possible financial situation” (0) to “the best possible financial situation” (10). We re-coded responses as: 8-10 (0), 4-7 (1), and 0-3 (2). Participants were asked whether they generally had enough money to meet their needs, and responses were coded as: more money than you need (0), just enough money (1), and not enough money (2). Finally, participants were asked about their difficulty paying monthly bills, and responses were coded as: not at all difficult (0), not very difficult (1), and somewhat or very difficult (2). We summed values across the four items to create the current SED score, with higher scores (range 0 to 8) reflecting greater current socioeconomic disadvantage.

**Table S1. Items Used to Create the Adverse Childhood Experience Score and Comparison Items Used in the Behavioral Risk Factor Surveillance System Adverse Childhood Experiences Module**

|  | **MIDUS** | | | |  | **BRFSS (2011-2014)^a^** | | |
| --- | --- | --- | --- | --- | --- | --- | --- | --- |
| **Category** | **Collection** | **Item(s)** | **Scoring** | **Prevalence^b^** |  | **Item(s)** | **Scoring** | **Prevalence** |
| Emotional abuse^c^ | M1, MR1  [in SAQ] | During your childhood, how often did your [mother or woman who raised you/father or man who raised you], do any of the things on List A to you?   - Insulted you or swore at you - Sulked or refused to talk to you - Stomped out of the room - Did or said something to spite you - Threatened to hit you - Smashed or kicked something in anger | Yes/1 = often, sometimes [from at least one parent]  No/0 = rarely, never, does not apply [from both parents]^d^ | 35.0% |  | How often did a parent or adult in your home ever swear at you, insult you, or put you down? | Yes/1 = once, more than once  No/0 = never | 34.4% |
| Physical abuse^c^ | M1, MR1  [in SAQ] | During your childhood, how often did your [mother or woman who raised you/father or man who raised you], do any of the things on List C to you?   - Kicked, bit, or hit you with a fist - Hit or tried to hit you with something - Beat you up - Choked you - Burned or scalded you | Yes/1 = often, sometimes, rarely [from at least one parent]  No/0 = never, does not apply [from both parents]^d^ | 20.9% |  | Before age 18, how often did a parent or adult in your home ever hit, beat, kick, or physically hurt you in any way? Do not include spanking. | Yes/1 = once, more than once  No/0 = never | 17.9% |
| Sexual abuse | M2, MR1  [in SAQ] | The following questions are about experiences you may have had at ANYTIME. Check the appropriate boxes next to any of the following experiences you have had. For those you checked, indicate how old you were, and if it affected you positively or negatively, both initially, and in the long run.^e^   - Sexually assaulted (e.g. forced sexual intercourse or other unwanted sexual contact)   - At what age(s) did this happen? | Yes/1 = checked sexually assaulted and happened at age <18 years | 6.2% |  | How often did anyone at least 5 years older than you or an adult,   - ever touch you sexually? - try to make you touch them sexually? - force you to have sex? | Yes/1 = once, more than once  No/0 = never | 11.6% |
| Parental separation or divorce | M2, MR1 [in SAQ] | The following questions are about experiences you may have had at ANYTIME. Check the appropriate boxes next to any of the following experiences you have had. For those you checked, indicate how old you were, and if it affected you positively or negatively, both initially, and in the long run.^e^   - Parents divorced   - At what age(s) did this happen? | Yes/1 = checked parents divorced and happened at age <18 years  **AND/OR**  Yes/1 = reported as a reason not living with biological parent in childhood: parents separated/divorced | 18.3% |  | Were your parents separated or divorced? | Yes/1 = yes  No/0 = no, parents not married | 27.6% |
|  | M1, MR1  [in phone] | Did you live with both of your biological parents up until you were 16?  IF NO-  Why didn't you live with your biological parents?   - Parents separated/divorced |  |  |  |  |  |  |
| Household alcohol or substance abuse | M2, MR1  [in phone] | When you were growing up, that is during your first 16 years, did you live with anyone who was a problem drinker or alcoholic? | Yes/1 = yes problem drinker or alcoholic  No/0 = no problem drinker or alcoholic  **AND/OR**  Yes/1 = checked one or both parents drank and happened at age <18 years  **AND/OR**  Yes/1 = checked one or both parents used drugs and happened at age <18 years | 24.1% |  | Did you live with anyone who was a problem drinker or alcoholic?  Did you live with anyone who used illegal street drugs or who abused prescription medications? | Yes/1 = yes  [to at least one question]  No/0 = no  [to both questions] | 27.6% |
|  | M2, MR1  [in SAQ] | The following questions are about experiences you may have had as a CHILD or TEENAGER. Check the appropriate boxes next to any of the following experiences you have had. For those you checked, indicate how old you were, and if it affected you, positively or negatively, both initially, and in the long run.^e^   - One or both parents drank so often it caused problems   - At what age(s) did this happen to you? - One or both parents used drugs so often it regularly caused problems   - At what age(s) did this happen to you? |  |  |  |  |  |  |

Note: Behavioral Risk Factor Surveillance System, BRFSS; Midlife in the United States, MIDUS; MIDUS 1 (1995-1996), M1; MIDUS 2 (2004-2006), M2; MIDUS Refresher 1 (2011-2014), MR1; self-administered questionnaire, SAQ. For the MIDUS 2 cohort, we used responses collected during MIDUS 1 (1995-1996) for some items, as indicated, if the items were not asked at MIDUS 2.

^a^ All items on BRFSS ask the respondent to consider the time period before 18 years of age. Centers for Disease Control and Prevention. Violence prevention: Behavioral Risk Factor Surveillance System ACE Data. US Department of Health and Human Services, 2020. <https://www.cdc.gov/violenceprevention/acestudy/ace_brfss.html>. Accessed October 1, 2020.

^b^ N=4344. Weighted prevalence.

^c^ The items assessing emotional and physical abuse asked respondents to indicate their experience of each, separately for their mother, father, brothers, sisters, and anybody else. For our analysis, we considered only the responses pertaining to mother and father in order to best align with items from BRFSS.

^d^ In instances in which the respondent reported a “no” for one parent but was missing a response for the other parent, we classified this as a “no” for both parents.

^e^ For alignment with BRFSS, we did not consider responses from the latter half of this question (…“and if it affected you positively or negatively, both initially, and in the long run”).

**Table S2. Comparison of Demographic Characteristics of MIDUS Participants Included and Not Included in the Analyses**

|  | **Included in analysis^b^** | |
| --- | --- | --- |
| **Characteristic^a^** | **Yes (N=4344),  % (95% CI)** | **No (N=1490), % (95% CI)** |
| Age, years |  |  |
| <30 | 4.5 (3.7, 5.5) | 8.0 (6.4, 10.0) |
| 30-39 | 17.1 (15.8, 18.6) | 24.6 (22.2, 27.1) |
| 40-49 | 23.9 (22.4, 25.4) | 28.6 (26.1, 31.2) |
| 50-59 | 24.6 (23.1, 26.1) | 19.6 (17.4, 22.0) |
| 60-69 | 18.3 (17.1, 19.5) | 12.6 (11.0, 14.4) |
| ≥70 | 11.7 (10.7, 12.7) | 6.6 (5.4, 8.0) |
| Gender |  |  |
| Female | 53.9 (52.1, 55.6) | 46.2 (43.3, 49.0) |
| Male | 46.1 (44.4, 47.9) | 53.8 (51.0, 56.7) |
| Highest level of education |  |  |
| ≤ High school/GED | 40.2 (38.4, 42.0) | 41.7 (38.8, 44.7) |
| Some college or technical school | 26.9 (25.4, 28.3) | 30.1 (27.7, 32.6) |
| College | 18.6 (17.5, 19.8) | 18.1 (16.3, 20.1) |
| ≥ Master’s degree | 14.3 (13.3, 15.4) | 10.1 (8.7, 11.6) |
| Major depressive episode |  |  |
| Yes | 13.7 (12.5, 15.0) | 15.3 (13.3, 17.5) |
| No | 86.3 (85.0, 87.5) | 84.7 (82.5, 86.7) |
| Anxiety disorder |  |  |
| Yes | 10.0 (8.9, 11.1) | 12.1 (10.3, 14.1) |
| No | 90.0 (88.9, 91.1) | 87.9 (85.9, 89.7) |

Note: Midlife in the United States, MIDUS.

^a^ Includes variables available from phone survey in MIDUS 2 and MIDUS Refresher, because these variables were available for all those included and not included in the analysis.

^b^ % (95% CI) = weighted percentages of sample. For those included in the analysis (were *not* missing data on the Adverse Childhood Experiences Score because they completed the self-administered questionnaire) the post-stratification weight variables used were A1SWGHT6 for MIDUS 2^5^ and RA1SWGHT6 for MIDUS Refresher (personal communication from Barry T. Radler, PhD of the MIDUS research team on July 16, 2020). For those not included in the study (were missing data on the Adverse Childhood Experiences Score because they did not complete the self-administered questionnaire), the post-stratification variables used were A1PWGHT6 for MIDUS 2 and RA1PWGHT6 for MIDUS Refresher. Percentages may not add to 100 due to rounding. Participants were missing data as follows: highest level of education (5 cases for those included and 4 cases for those not included in the analysis).

**Table S3. Relationships between Mental Health Conditions**

|  |  |  | **Prevalence of Another Mental Health Condition^b^** | | | |
| --- | --- | --- | --- | --- | --- | --- |
| **Mental Health Condition** | **Gender** | **No. (%)^a^** | **MDE,  No. (%)** | **PD, No. (%)** | **GAD,  No. (%)** | **Any Additional Condition,**  **No. (%)** |
| Major Depressive Episode (MDE) | All | 521 (13.7) | -- | 121 (24.9) | 97 (22.0) | 186 (39.2) |
|  | Males | 156 (9.0) | -- | 32 (23.9) | 25 (23.8) | 49 (38.8) |
|  | Females | 365 (17.8) | -- | 89 (25.4) | 72 (21.2) | 137 (39.4) |
|  |  |  |  |  |  |  |
| Panic Disorder (PD) | All | 289 (7.4) | 121 (46.2) | -- | 43 (19.4) | 132 (51.4) |
|  | Males | 74 (4.2) | 32 (51.6) | -- | 10 (21.4) | 34 (53.9) |
|  | Females | 215 (10.2) | 89 (44.4) | -- | 33 (18.6) | 98 (50.5) |
|  |  |  |  |  |  |  |
| Generalized Anxiety Disorder (GAD) | All | 126 (4.0) | 97 (76.0) | 43 (36.1) | -- | 108 (85.6) |
|  | Males | 33 (2.8) | 25 (75.2) | 10 (31.3) | -- | 27 (78.5) |
|  | Females | 93 (4.9) | 72 (76.3) | 33 (38.4) | -- | 81 (89.0) |
|  |  |  |  |  |  |  |
| Anxiety Disorder (AD) | All | 372 (10.0) | 186 (54.2) | 289 (74.5) | 126 (40.0) | 186 (54.2) |
|  | Males | 97 (6.1) | 49 (57.1) | 74 (68.1) | 33 (46.5) | 49 (57.1) |
|  | Females | 275 (13.2) | 137 (53.1) | 215 (77.0) | 93 (37.4) | 137 (53.1) |

^a^ No. (%) = unweighted n and weighted percentage with first condition.

^b^ No. (%) = unweighted n and weighted percentage among those with the first mental health condition (far left column) who also have at least one of the other mental health conditions. For example, in the “All” row for MDE, 24.9% of those with MDE also had PD.

**Table S4. Relationships between Categories of Adverse Childhood Experiences**

|  |  |  | **Prevalence of Exposure to Another Category of Adverse Childhood Experience^b^** | | | | | |
| --- | --- | --- | --- | --- | --- | --- | --- | --- |
| **Category of Adverse Childhood Experience** | **Gender** | **No. (%)^a^** | **Emotional abuse,**  **No. (%)** | **Physical abuse,**  **No. (%)** | **Sexual abuse,**  **No. (%)** | **Household alcohol or substance abuse,**  **No. (%)** | **Divorce- Separation, No. (%)** | **Any Additional Category,**  **No. (%)** |
| Emotional abuse | All | 1429 (35.0) | -- | 659 (46.1) | 142 (10.3) | 499 (36.6) | 305 (24.4) | 1017 (72.1) |
|  | Males | 633 (34.5) | -- | 320 (51.1) | 23 (3.3) | 207 (35.4) | 125 (22.4) | 446 (71.6) |
|  | Females | 796 (35.4) | -- | 339 (41.9) | 118 (16.1) | 292 (37.5) | 180 (26.2) | 571 (72.6) |
|  |  |  |  |  |  |  |  |  |
| Physical abuse* | All | 871 (20.9) | 659 (77.4) | -- | 93 (10.7) | 300 (36.7) | 178 (23.2) | 730 (85.4) |
|  | Males | 448 (23.6) | 320 (74.8) | -- | 19 (3.8) | 143 (36.0) | 86 (22.4) | 363 (84.3) |
|  | Females | 423 (18.5) | 339 (80.1) | -- | 74 (18.1) | 157 (37.6) | 92 (24.0) | 367 (86.5) |
|  |  |  |  |  |  |  |  |  |
| Sexual abuse* | All | 259 (6.2) | 142 (57.7) | 93 (35.7) | -- | 97 (37.2) | 69 (30.4) | 203 (80.9) |
|  | Males | 46 (2.3) | 24 (49.7) | 19 (39.0) | -- | 14 (28.8) | 9 (24.0) | 33 (73.7) |
|  | Females | 213 (9.6) | 118 (59.4) | 74 (35.1) | -- | 83 (38.9) | 60 (31.7) | 170 (82.4) |
|  |  |  |  |  |  |  |  |  |
| Household alcohol or substance abuse | All | 1007 (24.1) | 499 (53.1) | 300 (31.8) | 97 (9.6) | -- | 296 (32.8) | 685 (71.0) |
|  | Males | 421 (22.5) | 207 (54.4) | 143 (37.7) | 14 (3.0) | -- | 130 (34.5) | 291 (73.3) |
|  | Females | 586 (25.4) | 292 (52.2) | 157 (27.4) | 83 (14.7) | -- | 166 (31.5) | 394 (69.4) |
|  |  |  |  |  |  |  |  |  |
| Divorce-Separation | All | 690 (18.3) | 305 (46.7) | 178 (26.4) | 69 (10.3) | 296 (43.1) | -- | 480 (69.4) |
|  | Males | 304 (18.5) | 125 (41.7) | 86 (28.6) | 9 (3.0) | 130 (41.8) | -- | 204 (64.8) |
|  | Females | 386 (18.2) | 180 (51.0) | 92 (24.5) | 60 (16.7) | 166 (44.2) | -- | 276 (73.3) |

* *P* <.001 for chi-square test comparing the weighted percentage (prevalence) of the specific category of adverse childhood experience in males versus females.

^a^ No. (%) = unweighted n and weighted percentage with first category of adverse childhood experience.

^b^ No. (%) = unweighted n and weighted percentage among those with the first category of adverse childhood experience (far left column) who also have at least one of the other categories of adverse childhood experience. For example, in the “All” row for emotional abuse, 46.1% of those who experienced emotional abuse also experienced physical abuse.

**Table S5. Odds of Major Depressive Episode and Anxiety Disorder Associated with Two Risk Factors: Specific Adverse Childhood Experiences and Gender**

|  | **Major Depressive Episode** | | | | | |  | **Anxiety Disorder** | | | | | |
| --- | --- | --- | --- | --- | --- | --- | --- | --- | --- | --- | --- | --- | --- |
|  |  |  | **Model 1^b^** |  | **Model 2^c^** |  |  |  |  | **Model 1^b^** |  | **Model 2^c^** |  |
| **Adverse Childhood Experience and Gender** | **Prevalence of Disorder, Proportion (%)^a^** |  | **Odds Ratio**  **(95% CI)** |  | **Odds Ratio**  **(95% CI)** | ***P***  **Value^d^** |  | **Prevalence of Disorder, Proportion (%)^a^** |  | **Odds Ratio**  **(95% CI)** |  | **Odds Ratio**  **(95% CI)** | ***P***  **Value^d^** |
| Emotional Abuse^e^ |  |  |  |  |  |  |  |  |  |  |  |  |  |
| No | 277/2915 (10.9) |  | Reference |  | Reference | -- |  | 179/2951 (7.4) |  | Reference |  | Reference | -- |
| Yes | 244/1429 (19.1) |  | 1.94 (1.56, 2.41) |  | 1.56 (1.23, 1.96) | <.001 |  | 193/1429 (14.7) |  | 2.18 (1.70, 2.80) |  | 1.74 (1.33, 2.28) | <.001 |
| Gender |  |  |  |  |  |  |  |  |  |  |  |  |  |
| Males | 156/2009 (9.0) |  | Reference |  | Reference | -- |  | 97/2009 (6.1) |  | Reference |  | Reference | -- |
| Females | 365/2335 (17.8) |  | 2.19 (1.72, 2.78) |  | 2.24 (1.75, 2.88) | <.001 |  | 275/2335 (13.2) |  | 2.34 (1.75, 3.12) |  | 2.40 (1.79, 3.21) | <.001 |
| Physical Abuse^f^ |  |  |  |  |  |  |  |  |  |  |  |  |  |
| No | 376/3473 (12.4) |  | Reference |  | Reference | -- |  | 266/3473 (8.9) |  | Reference |  | Reference | -- |
| Yes | 145/871 (19.0) |  | 1.78 (1.40, 2.27) |  | 1.57 (1.20, 2.04) | .001 |  | 106/871 (13.9) |  | 1.78 (1.34, 2.36) |  | 1.50 (1.12, 2.02) | .007 |
| Gender |  |  |  |  |  |  |  |  |  |  |  |  |  |
| Males | 156/2009 (9.0) |  | Reference |  | Reference | -- |  | 97/2009 (6.1) |  | Reference |  | Reference | -- |
| Females | 365/2335 (17.8) |  | 2.27 (1.78, 2.88) |  | 2.32 (1.80, 2.98) | <.001 |  | 275/2335 (13.2) |  | 2.42 (1.81, 3.24) |  | 2.47 (1.84, 3.31) | <.001 |
| Sexual Abuse^g^ |  |  |  |  |  |  |  |  |  |  |  |  |  |
| No | 450/4085 (12.6) |  | Reference |  | Reference | -- |  | 310/4085 (8.9) |  | Reference |  | Reference | -- |
| Yes | 71/259 (30.9) |  | 2.58 (1.86, 3.59) |  | 2.23 (1.59, 3.14) | <.001 |  | 62/259 (26.1) |  | 2.99 (2.10, 4.26) |  | 2.56 (1.74, 3.78) | <.001 |
| Gender |  |  |  |  |  |  |  |  |  |  |  |  |  |
| Males | 156/2009 (9.0) |  | Reference |  | Reference | -- |  | 97/2009 (6.1) |  | Reference |  | Reference | -- |
| Females | 365/2335 (17.8) |  | 2.00 (1.58, 2.54) |  | 2.07 (1.61, 2.66) | <.001 |  | 275/2335 (13.2) |  | 2.10 (1.57, 2.80) |  | 2.16 (1.62, 2.90) | <.001 |
| Substance Abuse^h^ |  |  |  |  |  |  |  |  |  |  |  |  |  |
| No | 334/3337 (11.6) |  | Reference |  | Reference | -- |  | 235/3337 (8.1) |  | Reference |  | Reference |  |
| Yes | 187/1007 (20.4) |  | 1.92 (1.53, 2.40) |  | 1.64 (1.29, 2.08) | <.001 |  | 137/1007 (15.8) |  | 2.08 (1.60, 2.70) |  | 1.80 (1.37, 2.36) | <.001 |
| Gender |  |  |  |  |  |  |  |  |  |  |  |  |  |
| Males | 156/2009 (9.0) |  | Reference |  | Reference | -- |  | 97/2009 (6.1) |  | Reference |  | Reference | -- |
| Females | 365/2335 (17.8) |  | 2.15 (1.70, 2.73) |  | 2.23 (1.73, 2.86) | <.001 |  | 275/2335 (13.2) |  | 2.29 (1.72, 3.05) |  | 2.38 (1.78, 3.18) | <.001 |
| Divorce/Separation^i^ |  |  |  |  |  |  |  |  |  |  |  |  |  |
| No | 410/3654 (12.7) |  | Reference |  | Reference | -- |  | 286/3654 (8.9) |  | Reference |  | Reference | -- |
| Yes | 111/690 (18.6) |  | 1.60 (1.21, 2.10) |  | 1.19 (0.90, 1.57) | .229 |  | 86/690 (14.7) |  | 1.80 (1.32, 2.44) |  | 1.32 (0.97, 1.80) | .081 |
| Gender |  |  |  |  |  |  |  |  |  |  |  |  |  |
| Males | 156/2009 (9.0) |  | Reference |  | Reference | -- |  | 97/2009 (6.1) |  | Reference |  | Reference | -- |
| Females | 365/2335 (17.8) |  | 2.19 (1.73, 277) |  | 2.25 (1.76, 2.89) | <.001 |  | 275/2335 (13.2) |  | 2.35 (1.77, 3.12) |  | 2.42 (1.81, 3.23) | <.001 |

^a^ Prevalence is unadjusted. Proportion = number with the mental health disorder/number in the group defined by the adverse childhood experience (No/Yes) or gender (male or female). All numbers unweighted. Percentage = weighted 12-month prevalence of major depressive episode or anxiety disorder.

^b^ Model 1 is a logistic regression model with the mental health disorder (major depressive episode or anxiety disorder) as the dependent variable and ACE and gender as independent variables (*N* = 4344).

^c^ Model 2 is a logistic regression model with the mental health disorder (major depressive episode or anxiety disorder) as the dependent variable and ACE, gender, and 4 covariates (age, race, childhood socioeconomic disadvantage [SED] and current SED) as independent variables. *N* = 4275 after a listwise deletion of 69 participants (31 males and 38 females), who were missing data on race, childhood SED, or current SED.

^d^ *P* value for the Wald test, which was used to assess whether the addition of ACE or gender significantly improved the model fit over a model with 4 covariates and the other risk factor (ACE or gender).

^e^ Wald test was used to assess the addition of the gender x emotional abuse interaction term to Model 2 (assessing interaction in a multiplicative model). For MDE: F(1, 4274) = 2.93, *P* = .087. For AD: F(1, 4274) = 5.71, *P* = .017.

^f^ Wald test was used to assess the addition of the gender x physical abuse interaction term to Model 2 (assessing interaction in a multiplicative model). For MDE: F(1, 4274) = 1.82, *P* = .177. For AD: F(1, 4274) = 2.32, *P* = .128.

^g^ Wald test was used to assess the addition of the gender x sexual abuse interaction term to Model 2 (assessing interaction in a multiplicative model). For MDE: F(1, 4274) = 0.00, *P* = .979. For AD: F(1, 4274) = 1.85, *P* = .174.

^h^ Wald test was used to assess the addition of the gender x household alcohol or substance abuse interaction term to Model 2 (assessing interaction in a multiplicative model). For MDE: F(1, 4274) = 1.59, *P* = .208. For AD: F(1, 4274) = 0.12, *P* = .728. Substance abuse = household alcohol or substance abuse.

^i^ Wald test was used to assess the addition of the gender x divorce/separation interaction term to Model 2 (assessing interaction in a multiplicative model). For MDE: F(1, 4274) = 0.03, *P* = .852. For AD: F(1, 4274) = 0.03, *P* = .872.

**Table S6. Odds of Major Depressive Episode and Anxiety Disorder Associated with Specific Adverse Childhood Experiences, Stratified by Gender**

|  | **Major Depressive Episode** | | | | |  | **Anxiety Disorder** | | | | |
| --- | --- | --- | --- | --- | --- | --- | --- | --- | --- | --- | --- |
|  |  |  | **Model 1^b^** |  | **Model 2^c^** |  |  |  | **Model 1^b^** |  | **Model 2^c^** |
| **Adverse Childhood Experience** | **Prevalence of Disorder, Proportion (%)^a^** |  | **Odds Ratio**  **(95% CI)** |  | **Odds Ratio**  **(95% CI)** |  | **Prevalence of Disorder, Proportion (%)^a^** |  | **Odds Ratio**  **(95% CI)** |  | **Odds Ratio**  **(95% CI)** |
| Emotional Abuse – Males |  |  |  |  |  |  |  |  |  |  |  |
| No | 93/1376 (8.0) |  | Reference |  | Reference |  | 56/1376 (5.6) |  | Reference |  | Reference |
| Yes | 63/633 (10.9) |  | 1.40 (0.93, 2.11) |  | 1.19 (0.76, 1.85) |  | 41/633 (7.2) |  | 1.32 (0.80, 2.18) |  | 1.07 (0.63, 1.82) |
| Emotional Abuse – Females |  |  |  |  |  |  |  |  |  |  |  |
| No | 184/1539 (13.4) |  | Reference |  | Reference |  | 123/1539 (8.9) |  | Reference |  | Reference |
| Yes | 181/796 (25.9) |  | **2.27 (1.76, 2.92)** |  | **1.77 (1.35, 2.32)** |  | 152/796 (21.0) |  | **2.71 (2.03, 3.60)** |  | **2.20 (1.62, 2.99)** |
| Physical Abuse – Males |  |  |  |  |  |  |  |  |  |  |  |
| No | 112/1561 (8.4) |  | Reference |  | Reference |  | 70/1561 (5.9) |  | Reference |  | Reference |
| Yes | 44/448 (11.0) |  | 1.34 (0.85, 2.10) |  | 1.22 (0.76, 1.98) |  | 27/448 (7.0) |  | 1.19 (0.69, 2.08) |  | 1.07 (0.61, 1.88) |
| Physical Abuse – Females |  |  |  |  |  |  |  |  |  |  |  |
| No | 264/1912 (15.5) |  | Reference |  | Reference |  | 196/1912 (11.3) |  | Reference |  | Reference |
| Yes | 101/423 (27.7) |  | **2.08 (1.56, 2.78)** |  | **1.80 (1.31, 2.48)** |  | 79/423 (21.5) |  | **2.14 (1.54, 2.96)** |  | **1.78 (1.26, 2.51)** |
| Sexual Abuse – Males |  |  |  |  |  |  |  |  |  |  |  |
| No | 148/1963 (8.8) |  | Reference |  | Reference |  | 87/1963 (5.8) |  | Reference |  | Reference |
| Yes | 8/46 (16.9) |  | 2.10 (0.91, 4.85) |  | 2.21 (0.92, 5.32) |  | 10/46 (19.8) |  | **3.99 (1.80, 8.86)** |  | **4.74 (2.00, 11.23)** |
| Sexual Abuse – Females |  |  |  |  |  |  |  |  |  |  |  |
| No | 302/2122 (16.1) |  | Reference |  | Reference |  | 223/2122 (11.7) |  | Reference |  | Reference |
| Yes | 63/213 (33.8) |  | **2.66 (1.86, 3.82**) |  | **2.20 (1.52, 3.18)** |  | 52/213 (27.4) |  | **2.84 (1.93, 4.20)** |  | **2.35 (1.54, 3.59)** |
| Substance Abuse^d^ – Males |  |  |  |  |  |  |  |  |  |  |  |
| No | 106/1588 (8.0) |  | Reference |  | Reference |  | 66/1588 (5.1) |  | Reference |  | Reference |
| Yes | 50/421 (12.7) |  | **1.68 (1.09, 2.60)** |  | 1.34 (0.86, 2.11) |  | 31/421 (9.7) |  | **2.01 (1.19, 3.42)** |  | 1.68 (0.97, 2.90) |
| Substance Abuse^d^ – Females |  |  |  |  |  |  |  |  |  |  |  |
| No | 228/1749 (14.9) |  | Reference |  | Reference |  | 169/1749 (10.8) |  | Reference |  | Reference |
| Yes | 137/586 (26.3) |  | **2.03 (1.56, 2.65)** |  | **1.82 (1.37, 2.42)** |  | 106/586 (20.3) |  | **2.11 (1.57, 2.83)** |  | **1.85 (1.35, 2.54)** |
| Divorce/Separation – Males |  |  |  |  |  |  |  |  |  |  |  |
| No | 122/1705 (8.0) |  | Reference |  | Reference |  | 77/1705 (5.3) |  | Reference |  | Reference |
| Yes | 34/304 (13.4) |  | **1.78 (1.08, 2.94)** |  | 1.38 (0.84, 2.28) |  | 20/304 (9.8) |  | **1.94 (1.05, 3.58)** |  | 1.41 (0.77, 2.58) |
| Divorce/Separation – Females |  |  |  |  |  |  |  |  |  |  |  |
| No | 288/1949 (16.6) |  | Reference |  | Reference |  | 209/1949 (11.9) |  | Reference |  | Reference |
| Yes | 77/386 (23.1) |  | **1.50 (1.09, 2.08)** |  | 1.11 (0.80, 1.54) |  | 66/386 (19.0) |  | **1.74 (1.23, 2.45)** |  | 1.28 (0.89, 1.83) |

Note: Odds ratio in bold font indicates value >1.0 and 95% CI that excludes 1.0.

^a^ Prevalence is unadjusted. Proportion = number with the mental health disorder/number in the group defined by adverse childhood experience (No/Yes). All numbers unweighted. Percentage = weighted 12-month prevalence of major depressive episode or anxiety disorder.

^b^ Model 1 is a logistic regression model with the mental health disorder (major depressive episode or anxiety disorder) as the dependent variable and ACE as the independent variable. For males, *N* = 2009 and for females N = 2335.

^c^ Model 2 is a logistic regression model with the mental health disorder (major depressive episode or anxiety disorder) as the dependent variable and ACE and 4 covariates (age, race, childhood socioeconomic disadvantage [SED] and current SED) as independent variables. For males, *N* = 1978 after a listwise deletion of 31 participants who were missing data on race, childhood SED, or current SED. For females, *N* = 2297 after a listwise deletion of 38 participants who were missing data on race, childhood SED, or current SED.

^d^ Substance abuse = household alcohol or substance abuse.

**Table S7. Additive Interaction of Specific Adverse Childhood Experiences with Gender on Major Depressive Episode and Anxiety Disorder**

| **Specific Adverse Childhood Experiences** | **Gender** | **Major Depressive Episode** | | |  | **Anxiety Disorder** | | |
| --- | --- | --- | --- | --- | --- | --- | --- | --- |
|  |  | **Adjusted Prevalence %, (95% CI)^a^** | | **Interaction**  **Contrast (95% CI)^b^**  P_11_-P_10_- P_01_+ P_00_ |  | **Adjusted Prevalence %, (95% CI)^a^** | | **Interaction**  **Contrast (95% CI)^b^**  P_11_-P_10_- P_01_+ P_00_ |
|  |  | **No Abuse** | **Abuse** |  |  | **No Abuse** | **Abuse** |  |
| Emotional Abuse | Males | P_00_ = 8.6 (6.5, 10.6) | P_01_ = 9.8 (6.9, 12.6) | 22.6–14.4–9.8+8.6  **= 7.0 (1.9, 12.1)** |  | P_00_ = 6.0 (4.2, 7.8) | P_01_ = 6.3 (4.1, 8.6) | 18.2–9.7–6.3+6.0  **= 8.2 (3.7, 12.7)** |
|  | Females | P_10_ = 14.4 (12.3, 16.6) | P_11_ = 22.6 (19.5, 25.7) |  |  | P_10_ = 9.7 (7.9, 11.5) | P_11_ = 18.2 (15.3, 21.2) |  |
|  |  |  |  |  |  |  |  |  |
| Physical Abuse | Males | P_00_ = 8.6 (6.8, 10.5) | P_01_ = 10.2 (6.6, 13.9) | 24.6–15.9–10.2+8.6  **= 7.1 (0.7, 13.5)** |  | P_00_ = 6.0 (4.4, 7.7) | P_01_ = 6.4 (3.6, 9.2) | 18.5–11.7–6.4+6.0  **= 6.4 (0.9, 11.9)** |
|  | Females | P_10_ = 15.9 (14.0, 17.8) | P_11_ = 24.6 (20.1, 29.2) |  |  | P_10_ = 11.7 (10.0, 13.4) | P_11_ = 18.5 (14.4, 22.6) |  |
|  |  |  |  |  |  |  |  |  |
| Sexual Abuse | Males | P_00_ = 8.8 (7.2, 10.5) | P_01_ = 17.4 (5.9, 28.9) | 28.9–16.2–17.4+8.8  = 4.2 (-9.2, 17.5) |  | P_00_ = 5.8 (4.4, 7.2) | P_01_ = 20.4 (8.3, 32.5) | 22.8–11.9–20.4+5.8  = -3.6 (-17.4, 10.2) |
|  | Females | P_10_ = 16.2 (14.4, 18.1) | P_11_ = 28.9 (22.6, 35.3) |  |  | P_10_ = 11.9 (10.3, 13.4) | P_11_ = 22.8 (16.5, 29.1) |  |
|  |  |  |  |  |  |  |  |  |
| Household Alcohol or Substance Abuse | Males | P_00_ = 8.5 (6.6, 10.4) | P_01_ = 10.6 (7.1, 14.2) | 24.0–15.2–10.6+8.5  **= 6.6 (0.8, 12.4)** |  | P_00_ = 5.3 (3.8, 6.9) | P_01_ = 8.4 (5.2, 11.7) | 18.3–11.1–8.4+5.3  = 4.0 (-1.2, 9.3) |
|  | Females | P_10_ = 15.2 (13.2, 17.2) | P_11_ = 24.0 (20.3, 27.7) |  |  | P_10_ = 11.1 (9.4, 12.9) | P_11_ = 18.3 (14.9, 21.7) |  |
|  |  |  |  |  |  |  |  |  |
| Divorce/Separation | Males | P_00_ = 8.6 (6.9, 10.4) | P_01_ = 10.4 (6.3, 14.4) | 19.3–17.2–10.4+8.6  = 0.4 (-6.0, 6.8) |  | P_00_ = 5.7 (4.2, 7.1) | P_01_ = 7.6 (4.0, 11.2) | 15.4–12.5–7.6+5.7  = 1.0 (-4.7, 6.8) |
|  | Females | P_10_ = 17.2 (15.2, 19.2) | P_11_ = 19.3 (15.2, 23.4) |  |  | P_10_ = 12.5 (10.7, 14.2) | P_11_ = 15.4 (11.6, 19.2) |  |

Note: Interaction contrast in bold font indicates value >0 with 95% CI excluding 0 and is evidence of synergy between the two risk factors.

^a^ Weighted and adjusted 12-month prevalence of the outcome (major depressive episode or anxiety disorder) in the group defined by gender and specific adverse childhood experience (ACE). The adjusted prevalences (95% CI) were standardized to the distribution of covariates in the entire study population: age, race, childhood socioeconomic disadvantage (SED), and current SED.

^b^ The interaction contrast (95% CI) is calculated using the following formula: (P_11_-P_00_)-[(P_10_- P_00_)+(P_01_- P_00_)]. This formula can be simplified to: P_11_-P_10_- P_01_+ P_00._ In the formula, P represents the covariate-adjusted prevalence of the outcome and the subscripts 0 and 1 represent the groups defined by the presence (1) or absence (0) of one of the two risk factors. For example, P_00_ = male (0) with no ACEs (0) and P_11_ = female (1) with ACEs (1). Reported contrast values may vary from calculated values due to rounding.

**Figure S1. Adjusted Prevalence of Major Depressive Episode by Specific Adverse Childhood Experiences and Gender**


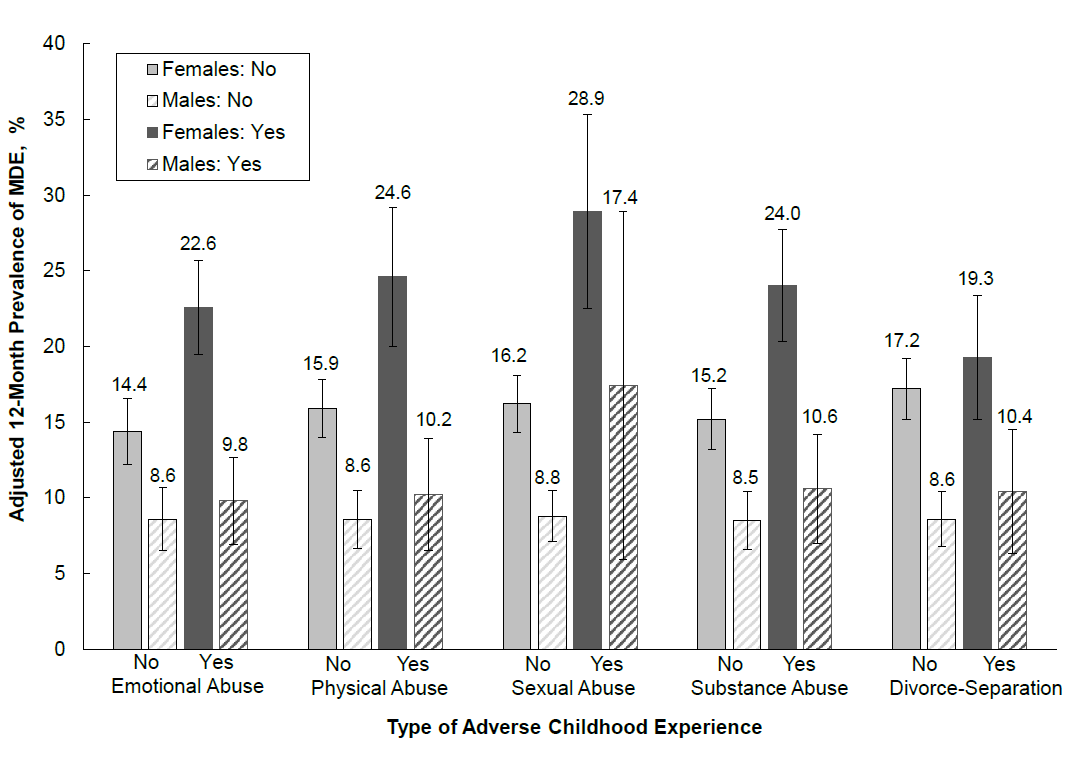


**eFigure 1 Legend:** Bars represent the adjusted 12-month prevalences of major depressive episode (MDE) by type of adverse childhood experience (ACE), and the uncertainty bars extend to the upper and lower bounds of the 95% confidence interval (CI). The adjusted prevalences (95% CI) were standardized to the distribution of covariates in the entire study population: age, race, childhood socioeconomic disadvantage, and current socioeconomic disadvantage. (Note: Substance abuse = household alcohol or substance abuse)

**Figure S2. Adjusted Prevalence of Anxiety Disorder by Specific Adverse Childhood Experiences and Gender**


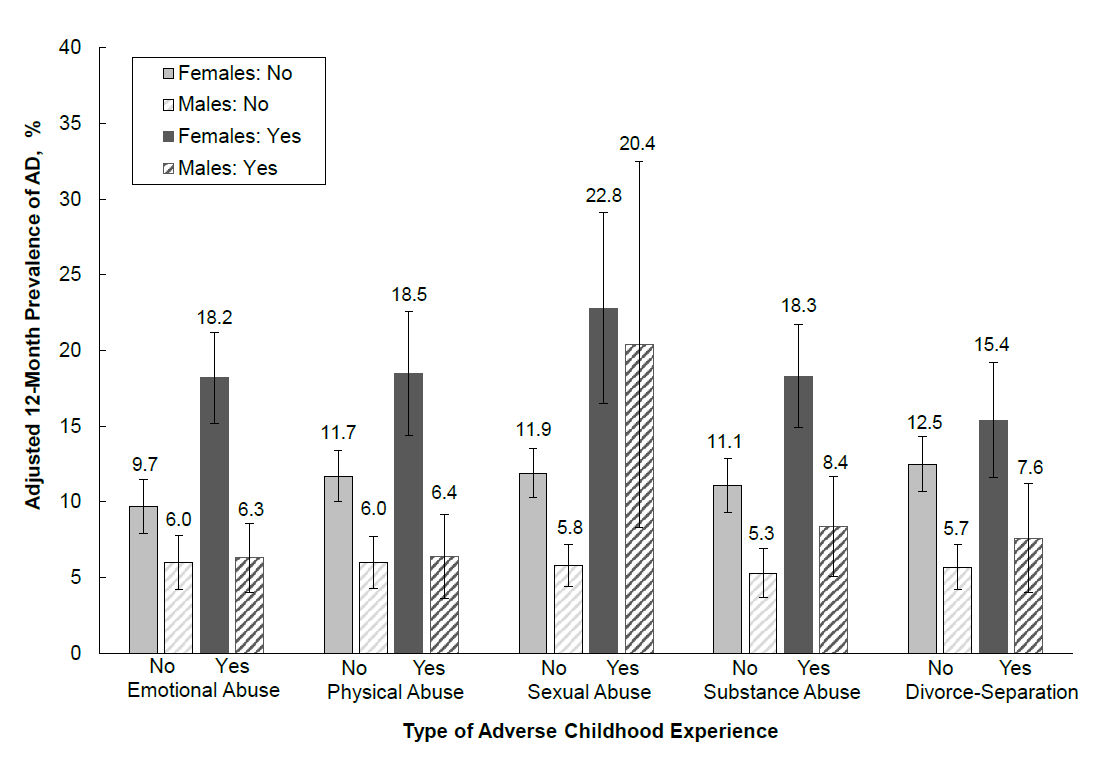


**eFigure 2 Legend:** Bars represent the adjusted 12-month prevalences of anxiety disorder (AD) by type of adverse childhood experience (ACE), and the uncertainty bars extend to the upper and lower bounds of the 95% confidence interval (CI). The adjusted prevalences (95% CI) were standardized to the distribution of covariates in the entire study population: age, race, childhood socioeconomic disadvantage, and current socioeconomic disadvantage. (Note: Substance abuse = household alcohol or substance abuse)

**SUPPLEMENTARY REFERENCES**

1. Lee C, Tsenkova VK, Boylan JM, Ryff CD. Gender differences in the pathways from childhood disadvantage to metabolic syndrome in adulthood: an examination of health lifestyles. *SSM Popul Health*. 2018;4:216-224. doi:10.1016/j.ssmph.2018.01.003

2. Tsenkova VK, Lee C, Boylan JM. Childhood socioeconomic disadvantage, occupational, leisure-time, and household physical activity, and diabetes in adulthood. *J Phys Act Health*. 2017;14(10):766-772. doi:10.1123/jpah.2016-0438

3. Boylan JM, Cundiff JM, Fuller-Rowell TE, Ryff CD. Childhood socioeconomic status and inflammation: psychological moderators among Black and White Americans. *Health Psychol*. 2020;39(6):497-508. doi:10.1037/hea0000866

4. Gruenewald TL, Karlamangla AS, Hu P, et al. History of socioeconomic disadvantage and allostatic load in later life. *Soc Sci Med*. 2012;74(1):75-83. doi:10.1016/j.socscimed.2011.09.037

5. Brim OG, Baltes PB, Bumpass LL, et al. *Midlife in the United States (MIDUS 1), 1995-1996: Documentation of Post-Stratification Weights Created at MIDUS 1 (ICPSR 2760)*. Ann Arbor, MI: Inter-University Consortium for Political and Social Research [distributor]; 2018. doi:10.3886/ICPSR02760.v18
